# Association Between Sports Participation and Sedentary Behavior During School Recess Among Brazilian Adolescents

**DOI:** 10.1515/hukin-2015-0023

**Published:** 2015-04-07

**Authors:** Diego Augusto Santos Silva, Roberto Jerônimo dos Santos Silva

**Affiliations:** 1 Federal University of Santa Catarina. Post-Graduate Program of Physical Education. Florianópolis, SC, Brazil.; 2 Federal University of Sergipe. Post-Graduate Program of Physical Education. Aracaju, SE, Brazil.

**Keywords:** adolescents, physical activity, sports, health, school

## Abstract

The aim of this study was to examine the association between sports participation and sedentary behavior during school recess among Brazilian adolescents. This study included 2,243 adolescents aged 13–18 years (16.2 ± 1.1), 62.2% females and 37.8% males, enrolled in public high schools in Aracaju, Northeastern Brazil. Sedentary behavior during school recess and sport participation was self-reported. Several factors were examined, including sex, age, skin color, socioeconomic status, maternal education and physical activity level. Sixty percent of adolescents had sedentary behavior during school recess and 57.7% of adolescents reported that they did not participate in any team sport. Additionally, adolescents who did not practice any team sport were 40% more likely (OR: 1.4, 95% CI: 1.1, 1.8) to be sedentary during school recess compared to those who participated in two or more team sports. It is recommended that schools encourage students to engage in sports activities and promote more physical activity during school recess to reduce the sedentary behavior and increase physical activity levels in youth.

## Introduction

Sedentary behavior during childhood is related to weight gain, being overweight or obese, poor performance in physical fitness tests, higher levels of blood pressure, total cholesterol and triglycerides (Tremblay et al., 2011a). In this sense, combating sedentary behavior is a public health priority (Tremblay et al., 2011b).

Children and adolescents spend a substantial proportion of their daily hours at school. Non-curricular time, such as school recess periods (recess and lunchtime) provide opportunities for children and adolescents to be physically active within the school environment (Jago and Baranowski, 2004). Recess periods may provide a unique opportunity during the school year to affect physical activity levels (Ridgers et al., 2012). Previous studies have suggested that recess may contribute between 5 and 40% toward the daily recommended physical activity (Ridgers et al., 2006) and 6 and 13% toward the total daily moderate-to-vigorous physical activity (Mota et al., 2005; Ridgers et al., 2009).

Organized youth sport is one of the most popular forms of leisure-time activities during adolescence (Kjønniksen et al., 2009). Sport is an important context for experiences related to identity and emotional development as well as a psychological competency and a positive peer context (Fredricks and Eccles, 2006). Additionally, youth participation in organized leisure-time activities is statistically associated with positive outcomes, such as healthier functioning, academic achievement, educational attainment, psychological adjustment, higher reports of engaging in vigorous activity, consuming fruit, and using a condom; and fewer reports of trying to lose weight, carrying a weapon, seriously considering suicide, attempting suicide, and smoking cigarettes (Kjønniksen et al., 2009; Taliaferro et al., 2010). In this sense, participation in organized sports may contribute to the development of lifelong physical activity (PA) habits (Kjønniksen et al., 2009).

Based on the benefits of practicing sports in adolescence (Taliaferro et al., 2010) and health hazards caused by sedentary behavior (Tremblay et al., 2011a), the hypothesis of this study was that adolescents who participated in organized sports were less sedentary during school recess than those who did not participate in organized sports. The aim of this study was to examine the association between sports participation and sedentary behavior during school recess among Brazilian adolescents.

## Material and Methods

This manuscript is based on the results of previously published research (Silva et al., 2014). This cross-sectional epidemiological study was carried out in the metropolitan area of the city of Aracaju, Sergipe, Brazil. The city of Aracaju is the capital of Sergipe, Brazil, and it has an estimated population of 571,149 inhabitants, poverty incidence of 27.5%, Gini index of 0.47 and Human Development Index of 0.794 (Brazilian Institute of Geography and Statistics, 2009; United Nations, 2000).

This study included 2,243 adolescents aged 13–18 years (16.2 ± 1.1), 62.2% females and 37.8% males. The adolescents were high school students from public schools of Aracaju and the metropolitan area. A high school in Brazil consists of three school grades (1st, 2nd and 3rd). Additional information on the sampling process can be verified in a previous publication (Silva et al., 2014). The study was approved by the Ethics Committee on Human Research of the Federal University of Sergipe, Brazil. All participants in the survey provided informed consent signed by their parents (if under 18 years of age) or themselves (≥18 years old).

All information was collected through questionnaires. Data collection was held in the second half of 2011. The questionnaires were administered in the classroom without the presence of teachers. The team of evaluators was composed of Physical Education graduate students who attended previous training for standardization of data collection procedures.

The dependent variable was sedentary behavior during school recess. Students were asked about their behavior during recess in the last seven days. The responses were as follows: 1) Sat down (talking, reading, or doing schoolwork); 2) Stood or walked around; 3) Ran or played a little bit; 4) Ran around and played quite a bit; and 5) Ran and played most of the time. This question is number three in the PAQ-C questionnaire (Crocker et al., 1997). This questionnaire has previously been used in research in Brazil (Silva et al., 2009; Silva et al., 2010). The behavior during recess was dichotomized into sedentary (response option 1) and active (response option 2, 3, 4 and 5). Recess in the schools that were investigated lasts 30 minutes and takes place after two and a half hours of class. After recess, students have another hour and forty minutes of class.

The independent variable was participation in team sports. The variable was assessed by the Brazilian version of the questionnaire used in the Youth Risk Behavior Surveillance System (YRBSS) in the United States. The YRBSS questionnaire was translated into Portuguese and validated for use in Brazil (Guedes and Lopes, 2010). Sports team participation was analyzed with the following item: During the past 12 months, on how many team sports did you play? The response to this item was ranked as “≥ 2 sports teams”, “1 sports team” and “no sports teams”. We chose this variable to understand whether practicing team sports in childhood and adolescence can provide a greater sense of collectiveness for young individuals (Fredricks and Eccles, 2006) in addition to stimulating the practice of physical activities. However, associations between individual sports, other types of physical activity and sedentary behavior during school recess need to be established for researchers to determine the best physical activities for adolescents.

The control variables were sex (male and female), age (in years; categorized into ≤ 16 years and 17–18 years), self-reported skin color (white, lighter skinned black, and dark skinned black), maternal schooling (≤ 8 years and > 8 years), economic level according to the Brazilian Association of Research Companies (2010) (high economic level, intermediate economic level, and low economic level), and physical activity level according to the Brazilian version of the questionnaire used in YRBSS (Guedes and Lopes, 2010). The physical activity level was assessed with the following question: During the past 7 days, on how many days were you physically active for at least 60 minutes a day? (Consider the time you spent in any kind of physical activity that increased your heart rate and made your breathing faster for some time). The response options were the following: No days, 1 day, 2 days, 3 days, 4 days, 5 days, 6 days, and 7 days. Participants who answered five or more days per week were classified as physically active and those who responded fewer than five days were classified as slightly physically active (Strong et al., 2005).

Data were double entered by two data-entry clerks and then analyzed in *Stata 11* (Stata Corp, College Station, Texas, USA). Descriptive and inferential statistics were applied. The linear trend and heterogeneity chi-square tests were used to assess the association between sedentary behavior during school recess and independent and control variables. In the crude and adjusted association analysis, the Wald test and binary logistic regression were used to estimate the odds ratios (OR) and 95% confidence intervals (CI). All variables were adjusted in the analysis, regardless of the *p*-value in crude analysis. The results were not stratified by sex because there was no interaction with this variable. Statistical tests were two-sided with a significance level of 5%.

## Results

Of the 2,243 respondents, 61% had sedentary behavior during school recess, and 57.7% reported that they had not participated in any team sport in the last 12 months. Table 1 shows the sample characteristics with respect to the demographic, socioeconomic, and physical activity variables.

According to Table 2, 65.2%, 57.9% and 51.7% of adolescents who had not participated or who participated in one or two team sports, respectively, had sedentary behavior during school recess (p <0.01). Furthermore, most boys and girls had sedentary behavior during school recess (p <0.01). There was no association between sedentary behavior during school recess and the other demographic, socioeconomic and physical activity variables (p >0.05).

**Figure 1:**
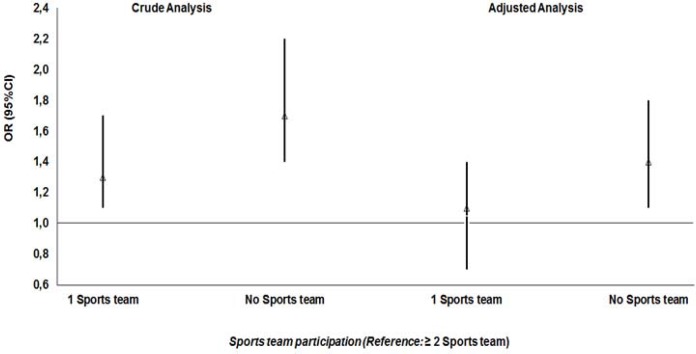
Estimated odds ratios and 95% confidence intervals in the association between sports team participation and sedentary behavior during school recess. OR: odds ratio; CI: confidence interval; ^*^Adjusted analysis for sex, age, socioeconomic status, skin color, maternal schooling and physical activity level.

Figure 1 shows the association between sedentary behavior during school recess and participation in team sports in the past 12 months. In crude analysis, we observed that young people who participated in a team sport (OR: 1.3, 95% CI: 1.1, 1.7) and those who did not participate in any team sport (OR: 1.7, 95% CI: 1.4, 2.2) were more likely to have a risk of sedentary behavior than adolescents who participated in two or more team sports. When adjusting the analysis by gender, age, socioeconomic status, skin color, maternal education, and physical activity level, the association between sedentary behavior during school recess and involvement in team sports was no longer significant (p > 0.05). However, the adjusted analysis found that adolescents who did not participate in any team sport were 40% more likely (OR: 1.4, 95% CI: 1.1, 1.8) to be sedentary during school recess compared to those who participated in two or more team sports.

## Discussion

The main finding of this study was that adolescents who participated in physical activities were less likely to have sedentary behavior during school recess. Furthermore, adolescents who did not participate in team sports were more likely to be sedentary during school recess.

The prevalence of sedentary adolescents during school recess was 61%. In England, 59.3% of students did not perform any physical activity during recess (Stratton et al., 2007). In California, USA, this percentage was 40.7% (McKenzie et al., 1997) and it was 48.5% in Hungary (Ridgers et al., 2009). These data show that many adolescents have sedentary behavior during school recess worldwide. This situation is concerning because there is evidence that young people with sedentary behavior in the school environment are more likely to remain sedentary throughout the day (Hohepa et al., 2009).

Sports activities can be considered an alternative to encouraging youth to participate in physical activities (Tremblay, 2012). The school environment that encourages physical activity through sports can be beneficial for both sports competitions and academic performance. Several studies have shown that physically active youth have better academic performance than sedentary ones (So, 2012; Syväoja et al., 2013). In this sense, the stimulus for physical activity in youth can be an alternative to exercise and engaging in other school activities.

The hypothesis of this study was confirmed, wherein adolescents who were not involved in any team sports throughout the year were more likely to be sedentary during school recess, regardless of sex, age, skin color, economic level, maternal education and physical activity level. The following are possible explanations for this finding: (i) young people who are involved in sports activities tend to be more physically active throughout the day, even in short-time intervals (Ridgers et al., 2012); (ii) young people who are involved in sports activities are more accepted by their peers at school (Seabra et al., 2013), and during school recess, they may engage in light, moderate or vigorous physical activities with these peers; and (iii) involvement in team sports results in greater body acceptance (Slater and Tiggemann, 2011), which situation may cause them to “show themselves” during school recess through participating in physical activities of different intensities. Such explanations require further investigation in future studies with a longitudinal design and qualitative approach.

Two previous studies have analyzed the types of physical activities that students perform during school recess and reported that those who practiced sports in this period were less likely to have sedentary behavior during school recess and higher physical activity levels throughout the day (Parrish et al., 2009; Ridgers et al., 2010). No previously published studies in the literature aimed to investigate the relationship between involvement in team sports and sedentary behavior during school recess, mediated by demographic and socioeconomic variables as well as physical activity level, which makes this the first study of its kind to analyze this association.

Demographic (gender, age, and skin color) and socioeconomic (maternal education and socioeconomic status) variables were controlled in the analysis of this study because previous studies have reported that these variables may influence sedentary behavior during the school break (Ridgers et al., 2012) and involvement in sports activities (Seabra et al., 2008). Ridgers et al. (2012) identified, through a systematic review, that boys were less sedentary during school breaks than girls; however, for the other variables (age, skin color, and socioeconomic status), the results were inconclusive. Regarding involvement in sports activities, Seabra et al. (2008) reported that population subgroups in Portugal that engaged in team sports were mostly boys and people with a high economic level.

The present study has limitations, such as the use of a questionnaire to measure the analyzed behaviors. The use of motion sensors to assess sedentary behavior in different contexts, including during school recess has increased in recent years (Colley et al., 2012; Ridgers et al., 2012). The divergence between the measurement instruments hinders more accurate comparisons between studies. The cross-sectional design is another limitation of this study because it does not allow for establishing a cause/effect relationship between participation in team sports and sedentary behavior during school recess.

Based on the present results, schools are recommended to encourage students to engage in sports activities, reducing the sedentary behavior and increasing the physical activity levels among youth. However, the stimulus for participating in competitive sports activities should be used with caution because some studies have reported that one of the reasons that children and adolescents withdraw from regular physical activity is a frustrating sporting experience (Seabra et al., 2013). In addition to sports practice, several initiatives to stimulate the involvement of adolescents in physical activities can be adopted, such as media advertisements, community programs and other strategies reported by Tremblay (2012). Additionally, schools should promote more physical activity during recess, encouraging adolescents to move through enjoyable activities and interactive games, such as the *exergames*, which are active video games (Simons et al., 2013).

## Conclusions

Six out of ten surveyed students had sedentary behavior during school recess. Students who were not engaged in team sports were more likely to have sedentary behavior during school breaks, regardless of the demographic and economic factors and physical activity level.

## Figures and Tables

**Table 1:** Sample distribution according to the variables.

**Variables**	**Sample**	
	
	**n**	**% (95%CI)**
*Total*	2,243	

*Sedentary behavior during school recess*		
*No*	868	39.0 (36.9, 41.0)
*Yes*	1,358	61.0 (58.9, 63.0)
*Sports team participation*		
≥ 2 Sports teams	388	17.4 (15.8, 18.9)
1 Sports team	556	24.9 (23.1, 26.7)
No Sports team	1,287	57.7 (55.6, 59.7)
*Sex*		
Male	836	37.8 (35.7, 39.7)
Female	1,377	62.2 (60.2, 64.2)
*Age (years)*		
≤16	1,272	57.0 (54.9, 59.0)
17–18	960	43.0 (40.9, 45.0)
*Skin color*		
White	301	15.4 (13.8, 17.0)
Lighter skinned black	1,195	61.2 (59.0, 63.3)
Dark skinned black	457	23.4 (21.5, 25.2)
*Socioeconomic status*		
High	525	23.7 (21.9, 25.4)
Intermediate	1,403	63.4 (61.3, 65.3)
Low	286	12.9 (11.5, 14.3)
*Maternal schooling*		
> 8 years	820	38.3 (36.2, 40.3)
≤ 8 years	1,321	61.7 (59.6, 63.7)
*Physical activity*		
Active	406	18.1 (16.5, 19.6)
Not very active	1,837	81.9 (80.3, 83.4)

CI: confidence interval.

**Table 2:** Prevalence of sedentary behavior during school recess according to the variables.

**Variables**	**Sedentary behavior during school recess**				
	
	**No**		**Yes**		***p***

	**n**	**% (95%CI)**	**n**	**% (95%CI)**	
*Sports team participation*					
≥ 2 Sports teams	185	48.3 (43.2, 53.3)	198	51.7 (46.7, 56.7)	<0.01
1 Sports team	231	42.1 (37.9, 46.2)	318	57.9 (53.7, 62.0)	
No Sports teams	443	34.8 (32.2, 37.4)	829	65.2 (62.5, 67.7)	
*Sex*					
Male	399	48.5 (45.1, 51.9)	423	51.5 (48.0, 54.9)	<0.01
Female	450	33.0 (30.5, 35.5)	912	67.0 (64.4, 69.4)	
*Age (years)*					
≤16	505	40.2 (37.5, 42.9)	752	59.8 (57.1, 62.5)	0.14
17–18	350	37.1 (33.9, 40.1)	594	62.9 (59.8, 66.0)	
*Skin color*					
White	102	34.5 (29.0, 39.9)	194	65.5 (60.0, 70.9)	0.16
Lighter skinned black	471	40.0 (37.1, 42.7)	707	60.0 (57.2, 62.8)	
Dark skinned black	184	40.8 (36.2, 45.3)	267	59.2 (54.6, 63.7)	
*Socioeconomic status*					
High	208	40.2 (36.0, 44.5)	309	59.8 (55.5, 64.0)	0.81
Intermediate	536	38.6 (36.1, 41.1)	852	61.4 (58.8, 63.9)	
Low	109	39.1 (33.3, 44.8)	170	60.9 (55.1, 66.6)	
*Maternal schooling*					
> 8 years	312	38.6 (35.2, 41.9)	496	61.4 (58.0, 64.7)	0.65
≤ 8 years	517	39.6 (36.9, 42.2)	789	60.4 (57.7, 63.0)	
*Physical activity*					
Active	163	40.9 (36.0, 45.6)	236	59.1 (54.3, 63.9)	0.40
Not very active	701	38.6 (36.3, 40.8)	1,115	61.4 (59.1, 63.6)	

CI: confidence interval.
